# Comparison of 11 staging classifications in carcinoma of the external auditory canal

**DOI:** 10.1007/s00405-025-09489-4

**Published:** 2025-06-02

**Authors:** Aniwat Berpan

**Affiliations:** Division of Radiation Oncology, Department of Radiology, Faculty of Medicine Vajira Hospital, Navamindradhiraj University, 681 Samsen Road, Dusit, Bangkok, 10300 Thailand

**Keywords:** AJCC 8th edition cutaneous carcinoma of the head and neck classification, Kinney classification, Modified Pittsburgh classification, Shih and Crabtree classification, Stell and McCormick classification

## Abstract

**Background and purpose:**

To compare overall survival (OS) differences across 11 different staging classifications.

**Materials and methods:**

Patient data were retrospectively collected. OS curves were estimated by Kaplan-Meier method. Hazard ratios (HR) were calculated by Cox regression. Concordance between HR of each system was evaluated by C-index. Absolute net reclassification improvement (NRI) was performed.

**Results:**

Thirty-five patients fitted study criteria with median follow-up of 27.3 months. 80% were diagnosed with squamous cell carcinoma. 14.3% had ECOG > 1. Eighteen patients underwent surgery, while 31 patients received radiotherapy. For AJCC 8th edition cutaneous carcinoma of the head and neck, 3-year OS of stage 1–3 and 4 was 26.7% (median OS of 0.8 years) and 64.1% (median OS of 4.3 years) (HR 0.33; 95%CI 0.09–1.2). 3-year OS of T1-3 and T4 in modified Pittsburgh classification were 65% and 55.4% with median OS of not reached and 3.1 years (HR 1.86; 95%CI 0.66–5.23). Regarding Kinney system, patients with stage 1–2 and 3 had 3-year OS of 67.1% and 45.5%, while median OS was 4.7 years and 1 year (HR 1.94; 95%CI 0.75–4.97). C-indices were 0.577, 0.523 and 0.569. NRI comparing Kinney with modified Pittsburgh classifications was 2.86%.

**Conclusion:**

Kinney’s may offer improved prognostic accuracy compared to other classifications.

**Supplementary Information:**

The online version contains supplementary material available at 10.1007/s00405-025-09489-4.

## Introduction

Carcinoma of the external auditory canal (EAC) is a rare malignancy. Among the various staging classifications available, the modified Pittsburgh system is the most widely accepted [1]. This system differs from the original classification [2] by redefining facial paralysis as a criterion for T4 rather than T3. Besides, Lavieille et al. subcategorized T4 in this classification into T4a, T4b and T4c in 1997 [3]. Due to the absence of a specific American Joint Committee on Cancer (AJCC) staging system for EAC cancer, staging classification for cutaneous carcinoma of the head and neck has often been adopted. Over the years, numerous other staging frameworks have been proposed. In 1976, Crabtree and colleagues classified EAC cancer into 2 stages, localized and extensive diseases. The former one is defined as disease confined to EAC and mastoid. Four years later Goodwin and Jesse introduced a three-tiered system based on the extent of involvement medially [4]. In 1985, Stell and McCormick published another classification including T1-3. While tumor confining within the organ of origin is described as T1-2, the definition of T3 is disease extension to dura, skull base, parotid gland or temporomandibular joint (TMJ). In 1989, Kinney marked stage 3 as cancer extension to dura, stylomastoid foramen or skull base. The author defined stage 1 as tumor limited in EAC and stage 2 as invasion of bone or middle ear (ME). One year after that, Shih and Crabtree defined stage 3 as disease extending to the parotid gland, neck, skull base and dura. Manolidis and colleagues proposed another four-stage system in 1998. They included involvement of TMJ, parotid gland, or infratemporal fossa into stage 2, ME, mastoid or facial nerve into stage 3 and dura, jugular bulb, sigmoid sinus, internal carotid artery or petrous apex into stage 4. Approximately two decades later, in 2021 systematic review was conducted by George et al. [5]. In this study, apart from primary tumor extension, cervical lymphadenopathy negatively influenced survival. Therefore, they proposed a new classification, which divides the disease into 4 stages (A-D), similar to the Kadish classification of esthesioneuroblastoma. Undoubtedly, cervical node involvement is incorporated in stage D. The definitions of each staging classification were demonstrated in Table S1 in the present article and Table 7 in reference 1.

Given the diverse staging systems and their potential implications for prognosis and treatment planning, the purpose of the present study was to compare survival differences among previous staging classifications which would facilitate standardization and guidance for management of EAC carcinoma.

## Materials and methods

### Patients

Data of patients with EAC cancer who were treated between January 2006 to December 2023 were retrospectively reviewed. Inclusion criteria were patients with early to locally advanced disease who underwent local treatment including surgery or radiotherapy. When survival details were not sufficiently reported, death certificates were obtained. Patients with metastatic disease, sarcoma, malignant melanoma or hematologic malignancy originating in EAC were excluded, as were individuals with a previous history of head and neck cancer, or incomplete data. This study was approved by the institutional review board.

### Procedures

After diagnosis of carcinoma of the EAC, resectability and operability were assessed. Surgery would be performed if patients were suitable for resection. Type of operation was generally determined by disease extension. Patients unsuitable for surgery were evaluated by radiation and medical oncologists for either definitive radiation with/without concurrent chemotherapy or preoperative chemotherapy. If surgery was deemed infeasible following chemotherapy, definitive radiotherapy was delivered. Postoperative radiation with/without concurrent chemotherapy was usually provided in case of positive surgical margin, extranodal extension, T3-4 disease (as per the modified Pittsburgh classification), multiple lymphadenopathy, perineural invasion or lymphovascular invasion. Concurrent chemoradiation was routinely given for gross disease, surgical margin involvement or extranodal extension. Chemotherapy is always a platinum-based regimen, with single-agent regimen normally used during concurrent chemoradiotherapy, while doublet regimen always for neoadjuvant chemotherapy. Platinum-based chemotherapy with fluorouracil was mainly prescribed, followed by platinum-based chemotherapy with paclitaxel.

For radiotherapy, the technique and dose depended on machine availability at that time of treatment, patient’s performance status, disease characteristics and preference of the radiation oncologist. There were three radiation techniques composed of two-dimensional (2D), three-dimensional conformal (3DCRT) and intensity-modulated radiation (IMRT). Treatment planning of 3DCRT and IMRT was determined by target volume contouring. Radiation dose for R0-1 resections ranged from 50 to 66 Gy in 25–33 fractions, while 70 Gy in 33–35 fractions was generally given in R2 resection or definitive radiotherapy.

Disease staging was determined through physical examinations, imaging studies including computed tomography (CT), magnetic resonance imaging (MRI) and positron emission tomography (PET), surgical findings and pathological reports.

### Statistical analysis

Overall survival (OS) was calculated from the initiation of cancer treatment, which could be surgery, radiation or neoadjuvant chemotherapy to death from any causes. Patients without any events were censored at their last follow-up. Survival curves were constructed by the Kaplan-Meier method. Analysis of the differences between survival curves was performed with the log-rank test. Cox proportional hazards regression was used to estimate hazard ratios (HR). Harrell’s concordance index (C-index) was calculated to evaluate concordance between HR of each system. Absolute net reclassification improvement (NRI) was done to compare the performance of the best staging classification with modified Pittsburgh classification. All statistical tests were considered statistically significant at *p*-value ≤ 0.05. PASW Statistics (SPSS) 28.0 (SPSS Inc., Chicago, IL., USA) and Stata/SE, release 17 (StataCorp LP, College Station, TX, USA) were used for the analysis.

## Results

Of 45 patients evaluated for eligibility, 35 met the inclusion criteria. Ten patients were excluded due to reasons including lack of cancer treatment (*n* = 3), primary cancer originating in the pinna (*n* = 4), a history of nasopharyngeal or supraglottic cancer (*n* = 2), or incomplete data (*n* = 1). The mean age was 58.3 years (standard deviation 15.6). There were 20 female patients. 14.3% had an ECOG score more than one. Squamous cell carcinoma (SCC) was diagnosed in 28 patients. Four and three patients had adenocarcinoma and adenoid cystic carcinoma, respectively.

Eighteen patients received surgery, with procedures ranging from excision in 2 patients, mastoidectomy in 9 patients, subtotal petrosectomy in 1 patients as well as lateral and subtotal temporal bone resections in 3 and 2 patients, respectively. Parotidectomy and cervical lymph node dissection were done in 6 and 3 patients, respectively. While radiotherapy was performed in 31 patients, with techniques comprising 3DCRT in 61.3%, IMRT in 22.6%, and 2D techniques in 16.1%. Table 1 shows the patient and treatment characteristics of eligible patients.

**Table 1:** Characteristics of the patients at baseline

**Characteristics**	**(** ***n*** ** = 35)**
Age ≥ 65 years	12 (34.3)
Male	15 (42.9)
Smoking history	8 (22.9)
**ECOG score**	
0	24 (68.6)
1	6 (17.1)
2	4 (11.4)
3	1 (2.9)
HIV infection	2 (5.7)
**Squamous cell carcinoma**	28 (80)
**Histologic differentiation**	
Well differentiated	10 (28.6)
Moderately differentiated	18 (51.4)
Poorly differentiated	3 (8.6)
Neoadjuvant chemotherapy	3 (8.6)
**Surgery**	18 (51.4)
**Resection status**	
R0 resection	5 (27.8)
R1 resection	2 (11.1)
R2 resection	5 (27.8)
**Radiotherapy**	31 (88.6)
**Radiotherapy technique**	
2D	5 (16.1)
3DCRT	19 (61.3)
IMRT	7 (22.6)
Elective nodal irradiation	6 (19.4)
Concurrent chemoradiation	21 (67.7)
Postoperative radiotherapy	14 (40)

ECOG denotes Eastern Cooperative Oncology Group

**Fig. 1:**
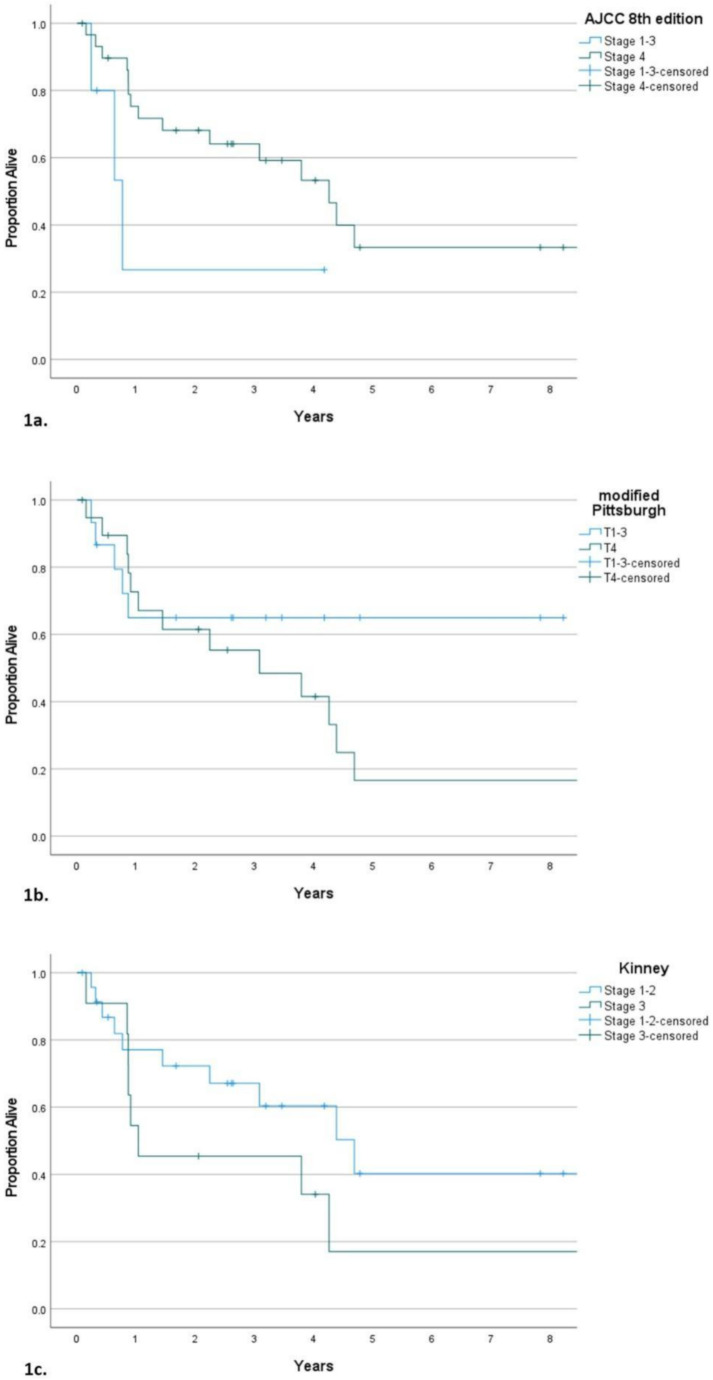
Overall survival of patients with carcinoma of the external auditory canal classified by AJCC 8th edition cutaneous carcinoma of the head and neck (**1a**), modified Pittsburgh (**1b**) and Kinney classifications (**1c**)

The median follow-up time was 27.3 months. In accordance with the George classification, 3-year OS rates for stage A-B, C and D were 65.5%, 42.9% and 60%, respectively. Median OS was 4.4 years, 1 year and not reached (HR 2.21; 95%CI 0.78–6.28 for stage C and HR 0.67; 95%CI 0.15–3.1 for stage D). Rates of OS at 3 years for stage 1–3 and 4 of the AJCC 8th edition staging system of cutaneous carcinoma of the head and neck were 26.7% and 64.1%, respectively with median OS of 0.8 and 4.3 years (HR 0.33; 95%CI 0.09–1.2). Regarding the modified Pittsburgh classification, patients with T1-3 and T4 had 3-year OS rates of 65% and 55.4%, respectively. Median OS was not reached and 3.1 years (HR 1.86; 95%CI 0.66–5.23). For the Manolidis system, 50%, 67% and 55.6% were OS rates at 3 years of stage 1–2, 3 and 4, respectively. Median OS was 0.8, 4.4 and 3.8 years (HR 0.48; 95%CI 0.14–1.7 for stage 3 and HR 0.85; 95%CI 0.24–3.1 for stage 4). According to the Lavieille classification, patients defined as T1-3, T4a, T4b and T4c had OS rates at 3 years of 61.6%, 50%, 33.3% and 50%, respectively with median OS of 4.4, 3.1, 1.4 and 1 years (HR 0.52; 95%CI 0.06–4.11 for T4a, HR 1.27; 95%CI 0.27–5.9 for T4b and HR 1.92; 95%CI 0.66–5.6 for T4c). For the Pittsburgh staging system, 3-year OS rates of T1-3 and T4 were 61.6% and 57.1%, respectively. Median OS was 4.4 and 3.1 years (HR 1.34; 95%CI 0.53–3.41). OS rates at 3 years of stage 1–2 and 3 in accordance with classification proposed by Shih and Crabtree were 64.6% and 54.7%, respectively. Median OS was 4.7 and 3.1 years (HR 1.53; 95%CI 0.58–4.03). According to the Kinney staging system, stage 1–2 and 3 had 3-year OS rates of 67.1% and 45.5%, respectively. 4.7 and 1 years were their median OS (HR 1.94; 95%CI 0.75–4.97). Patients with T1-2 and T3 proposed by Stell and McCormick had OS rates at 3 years of 64.6% and 54.7%, respectively with median OS of 4.7 and 3.1 years (HR 1.53; 95%CI 0.58–4.03). In accordance with the classification proposed by Goodwin and Jesse, 3-year OS rates of T1-2 and T3 were 55.6% and 60.9%, respectively. Median OS was 4.7 and 3.8 years (HR 0.7; 95%CI 0.25–1.97). Finally, for localized and extensive stages proposed by Crabtree, their 3-year OS rates were 60% and 59.2%, respectively with median OS of not reached and 3.8 years (HR 1; 95%CI 0.09–1.2). C-indices were 0.520, 0.577, 0.523, 0.478, 0.538, 0.516, 0.536, 0.569, 0.536, 0.551 and 0.487, respectively. Additionally, absolute NRI comparing Kinney with modified Pittsburgh classifications was 2.86% (95%CI -0.33 to 0.41) (Table 2). To evaluate prognostic ability of lymph node metastases, regional lymphadenopathy (parotid plus cervical lymph nodes) and cervical lymphadenopathy were included in the analyses which demonstrated HR of 0.87 (95%CI 0.28–2.64) and 0.55 (95%CI 0.13–2.41), respectively (Table 3; Fig. 1 and Figure S1-11).

**Table 2:** Net reclassification improvement for overall survival: Kinney versus modified Pittsburgh classifications

	modified Pittsburgh	Kinney		
		Stage 1–2	Stage 3	Total
**Died during follow-up**	**T1-3**	10	0	10
	**T4**	4	3	7
	**Total**	14	3	17
**Survived during follow-up**	**T1-3**	4	1	5
	**T4**	6	7	13
	**Total**	10	8	18

**Table 3:** Overall survival and Harrell’s concordance index (C-index) across various staging classifications

Classification	(*n* = 35)	3-year OS (%)	Median OS (years)	*p*-value	HR (95% CI)	C-index (95% CI)
**George**				0.21		0.520 (0.412–0.745)
Stage A-B	23 (65.7)	65.5	4.4		1	
Stage C	7 (20)	42.9	1		2.21 (0.78–6.28)	
Stage D	5 (14.3)	60	NR		0.67 (0.15–3.1)	
**AJCC 8th edition cutaneous carcinoma of the head and neck**						0.577 (0.357–0.594)
Stage 1–3	5 (14.3)	26.7	0.8	0.08	1	
Stage 4	30 (85.7)	64.1	4.3		0.33 (0.09–1.2)	
T1-3	6 (17.1)	20.8	0.8	0.048	1	0.589 (0.350–0.637)
T4	29 (82.9)	66.5	4.3		0.57 (0.32–1.02)	
**modified Pittsburgh**				0.23		0.523 (0.494–0.816)
T1-3	15 (42.9)	65	NR		1	
T4	20 (57.1)	55.4	3.1		1.86 (0.66–5.23)	
**Manolidis**				0.42		0.478 (0.407–0.763)
Stage 1–2	9 (25.7)	50	0.8		1	
Stage 3	17 (48.6)	67	4.4		0.48 (0.14–1.7)	
Stage 4	9 (25.7)	55.6	3.8		0.85 (0.24–3.1)	
**Lavieille**				0.51		0.538 (0.440–0.782)
T1-3	20 (57.1)	61.6	4.4		1	
T4a	4 (11.4)	50	3.1		0.52 (0.06–4.11)	
T4b	3 (8.6)	33.3	1.4		1.27 (0.27–5.9)	
T4c	8 (22.9)	50	1		1.92 (0.66–5.6)	
**Pittsburgh**				0.53		0.516 (0.407–0.740)
T1-3	20 (57.1)	61.6	4.4		1	
T4	15 (42.9)	57.1	3.1		1.34 (0.53–3.41)	
**Shih and Crabtree**				0.38		0.536 (0.402–0.739)
Stage 1–2	16 (45.7)	64.6	4.7		1	
Stage 3	19 (54.3)	54.7	3.1		1.53 (0.58–4.03)	
**Kinney**				0.16		0.569 (0.483–0.785)
Stage 1–2	24 (68.6)	67.1	4.7			
Stage 3	11 (31.4)	45.5	1		1.94 (0.75–4.97)	
**Stell and McCormick**				0.38		0.536 (0.372–0.709)
T1-2	15 (42.9)	64.6	4.7		1	
T3	20 (57.1)	54.7	3.1		1.53 (0.58–4.03)	
**Goodwin and Jesse**				0.5		0.551 (0.354–0.662)
T1-2	10 (28.6)	55.6	4.7		1	
T3	25 (71.4)	60.9	3.8		0.7 (0.25–1.97)	
**Crabtree**				1		0.487 (0.413–0.652)
Stage 1	5 (14.3)	60	NR		1	
Stage 2	30 (85.7)	59.2	3.8		1 (0.23–4.47)	
**Regional lymphadenopathy**				0.8		0.501 (0.321–0.574)
No	27 (77.1)	63	4.3		1	
Yes	8 (22.9)	50	1.4		0.87 (0.28–2.64)	
**Cervical lymphadenopathy**				0.42		0.524 (0.348–0.587)
No	30 (85.7)	59.6	3.8		1	
Yes	5 (14.3)	60	NR		0.55 (0.13–2.41)	

AJCC denotes American Joint Committee on Cancer, NR Not reach, OS Overall survival

## Discussion

Staging plays a critical role in stratifying patients based on disease extent, guiding optimal management strategies, and ultimately influencing therapeutic outcomes. An effective classification system is therefore essential. The findings of the present study indicate that the Kinney staging classification might outperform others, as evidenced by its superior concordance and the appropriate direction and magnitude of differentiation in mortality risk between lower and higher stages, as well as net reclassification improvement.

Ten patients were defined as T4 in the modified Pittsburgh classification but not as stage 3 in the Kinney system. These included four with facial paresis, four with extensive soft tissue involvement and two with cochlear extension. Notably, nearly half of the patients categorized as stage 2 in the Kinney classification were assigned to T4 in the modified Pittsburgh system solely due to facial paresis. This discrepancy may explain why the modified Pittsburgh classification appeared less accurate than the Kinney system. Contrary to the findings of the present study, a systematic review by Higgins et al., which included 348 subjects, reported significantly worse OS among patients with facial paralysis compared to those without it [6]. Regarding the Kinney system, all patients classified as stage 3 had dura or skull base involvement, with or without stylomastoid foramen involvement. This suggested that dura and skull base invasion are the primary factors significantly impacting survival, while other factors might have a less substantial effect.

Regional lymph node metastasis is widely recognized as a poor prognostic factor in various cancers including EAC cancer. In multivariable analysis, conducted by Morris and colleagues, regional nodal metastasis was identified as an independent factor associated with OS (HR 4.67; 95%CI 1.42–15.36). Distinctively about half of the patients had histology of SCC [7]. Additionally, a study by Morita et al. found lymph node metastasis to be a significantly poor prognostic factor for OS in univariable analysis (HR 6.2; 95%CI 5.05–70.6). However, in multivariable analysis, lymph node metastasis did not remain significant, with T classification based on the modified Pittsburgh staging system as the only independent predictor of survival [8]. Inconsistent with the present study, neither regional nor cervical lymphadenopathy was a significant prognostic factor. This discrepancy might be attributed to the higher proportion of patients with T4 disease in the present study (57%) compared to the Morita’s study (21%). As a result, the influence of T staging might have outweighed the impact of lymphadenopathy on mortality risk.

In general, lateral temporal bone resection is indicated for cases with osseous EAC involvement. If the disease extends further but includes limited dural invasion (defined as less than 1 centimeter invasion), subtotal temporal bone resection can still achieve satisfactory surgical margins. However, in case of the disease progressing beyond this extent, for instance, with widespread dural invasion, internal carotid encasement, petrous apex extension or intraparenchymal involvement, obtaining adequate margin becomes infeasible, necessitating a shift to radiation treatment. While the staging classifications evaluated in the present study provide insights into prognosis, none reliably guide optimal treatment selection. Nevertheless, these potentially guide treatment adjustments. For instance, early-stage patients may benefit from reduced resected areas or radiation fields, whereas advanced-stage patients might require neoadjuvant or adjuvant therapies. In addition, beyond traditional clinicopathological staging, which relies on diagnostic imaging, operative findings, and surgical specimens, molecular classifications based on specific genetic, epigenetic or proteomic profiles hold promise. These approaches could improve patient stratification and facilitate treatment optimization, particularly in the context of personalized medicine.

In conclusion, among these systems, the Kinney classification possibly demonstrated superior performance, suggesting its potential as a more reliable tool for survival prediction. Future prospective studies with larger cohorts and the integration of molecular profiling are warranted to validate these results and optimize staging for EAC carcinoma.

## Electronic supplementary material

Below is the link to the electronic supplementary material.

Supplementary Material 1

Supplementary Material 2

## Data Availability

Research data are stored in an institutional repository and will be shared upon request to the corresponding author.

## References

[1] Moody SA, Hirsch BE, Myers EN (2000) Squamous cell carcinoma of the external auditory Canal: an evaluation of a staging system. Am J Otol 21(4):582–588 10912706

[2] Arriaga M, Curtin H, Takahashi H et al (1990) Staging proposal for external auditory meatus carcinoma based on preoperative clinical examination and computed tomography findings. Ann Otol Rhinol Laryngol 99(9 Pt 1):714–721 2396807 10.1177/000348949009900909

[3] Lavieille J, Reyt E, Boulat E et al (1997) Cancers du conduit auditif externe et de L’oreille Moyenne: indications chirurgicales et nouvelles classification. JFORL 46:357Y61

[4] Goodwin WJ, Jesse RH (1980) Malignant neoplasms of the external auditory Canal and Temporal bone. Arch Otolaryngol 106(11):675–679 6252880 10.1001/archotol.1980.00790350017006

[5] George M, Borsotti F, Gereige R et al (2021) A systematic review of the primary squamous cell carcinoma of the external auditory Canal: survival outcome based on T-staging and proposal of a new classification. J Laryngol Otol 135(2):96–103 33568243 10.1017/S0022215121000323

[6] Higgins TS, Antonio SA (2010) The role of facial palsy in staging squamous cell carcinoma of the Temporal bone and external auditory Canal: a comparative survival analysis. Otol Neurotol 31(9):1473–1479 20930655 10.1097/MAO.0b013e3181f7ab85

[7] Morris LG, Mehra S, Shah JP et al (2012) Predictors of survival and recurrence after Temporal bone resection for cancer. Head Neck 34(9):1231–1239 21953902 10.1002/hed.21883 PMC4126564

[8] Morita S, Homma A, Nakamaru Y et al (2016) The outcomes of surgery and chemoradiotherapy for Temporal bone Cancer. Otol Neurotol 37(8):1174–1182 27466887 10.1097/MAO.0000000000001152

