# Supplementary material for: Comparison of 11 staging classifications in carcinoma of the external auditory canal
Source: Eur Arch Otorhinolaryngol. 2025 Jun 2;282(9):4765–71. doi: 10.1007/s00405-025-09489-4 (PMC12423134; doi:10.1007/s00405-025-09489-4)

Figure S1. Overall survival of patients with carcinoma of the external auditory canal classified by George classification.


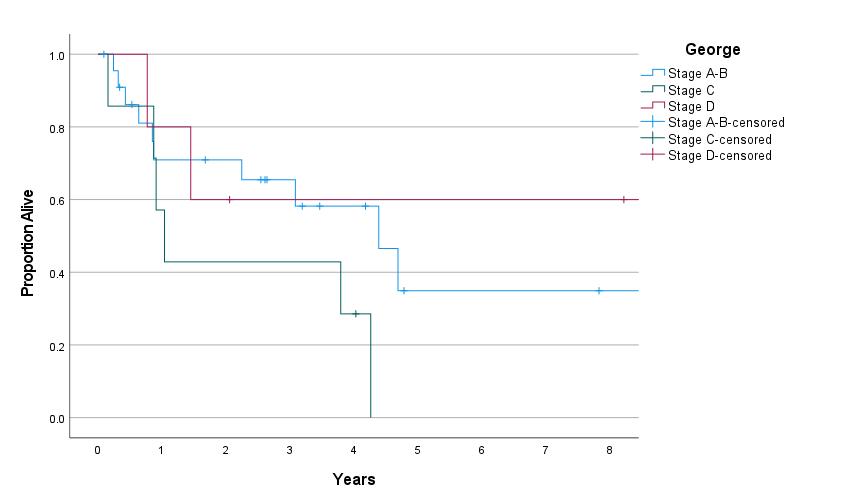


Figure S2. Overall survival of patients with carcinoma of the external auditory canal classified by T staging of AJCC 8th edition cutaneous carcinoma of the head and neck classification.


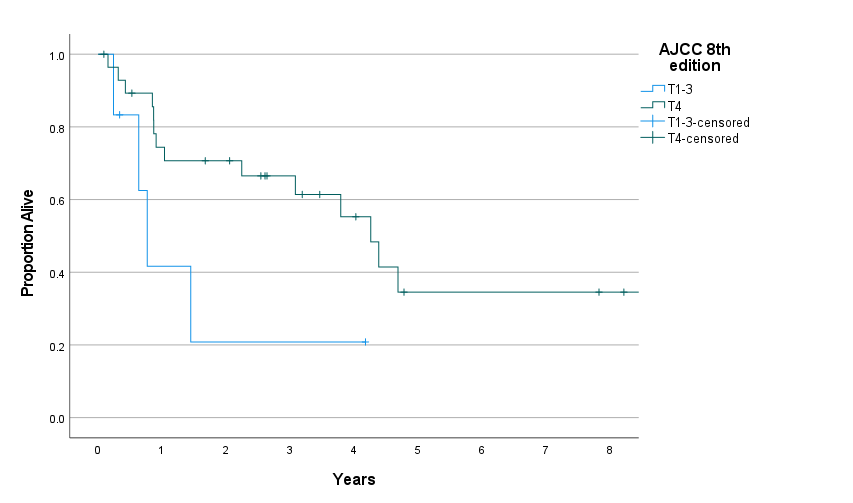


Figure S3. Overall survival of patients with carcinoma of the external auditory canal classified by Manolidis classification.


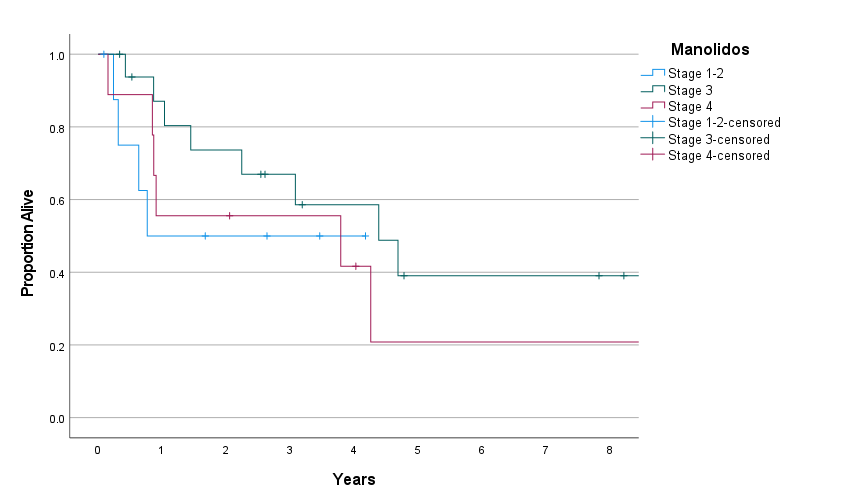


Figure S4. Overall survival of patients with carcinoma of the external auditory canal classified by Lavieille classification.
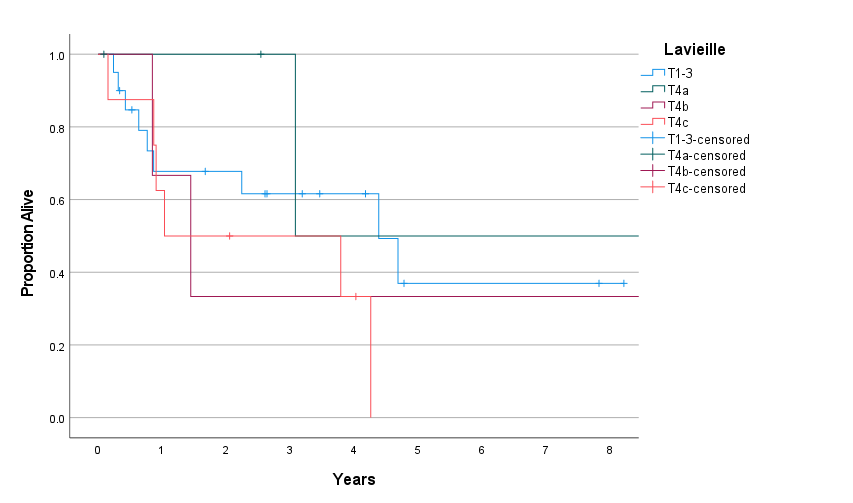


Figure S5. Overall survival of patients with carcinoma of the external auditory canal classified by Pittsburgh classification.


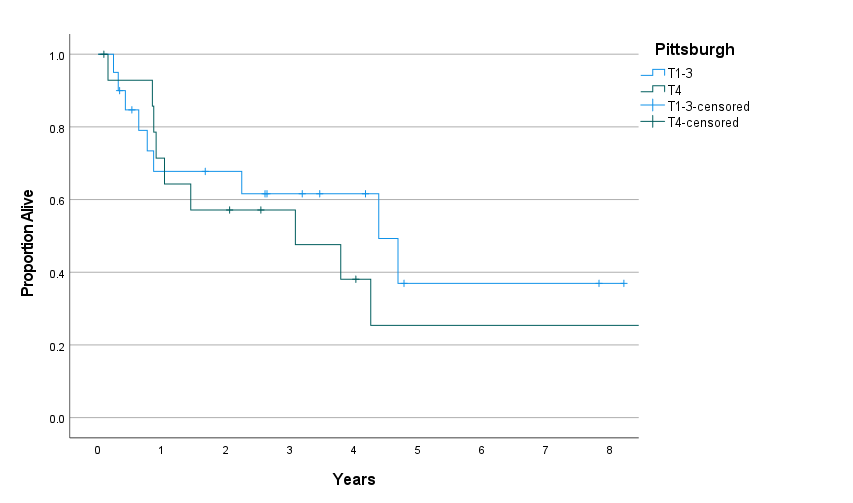
Figure S6. Overall survival of patients with carcinoma of the external auditory canal classified by Shih and Crabtree classification.


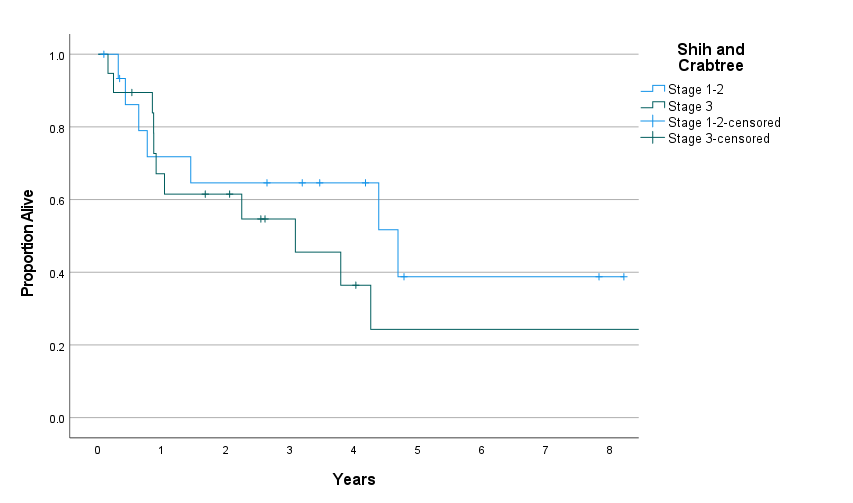
Figure S7. Overall survival of patients with carcinoma of the external auditory canal classified by Stell and McCormick classification.


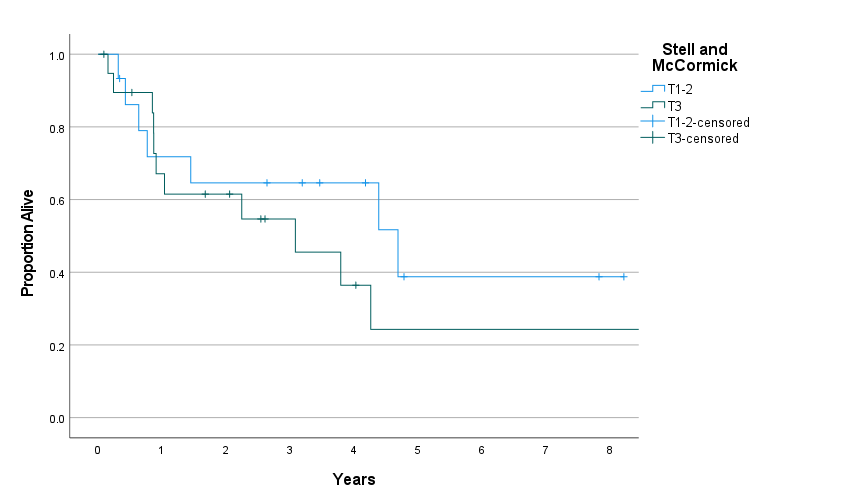
Figure S8. Overall survival of patients with carcinoma of the external auditory canal classified by Goodwin and Jesse classification.


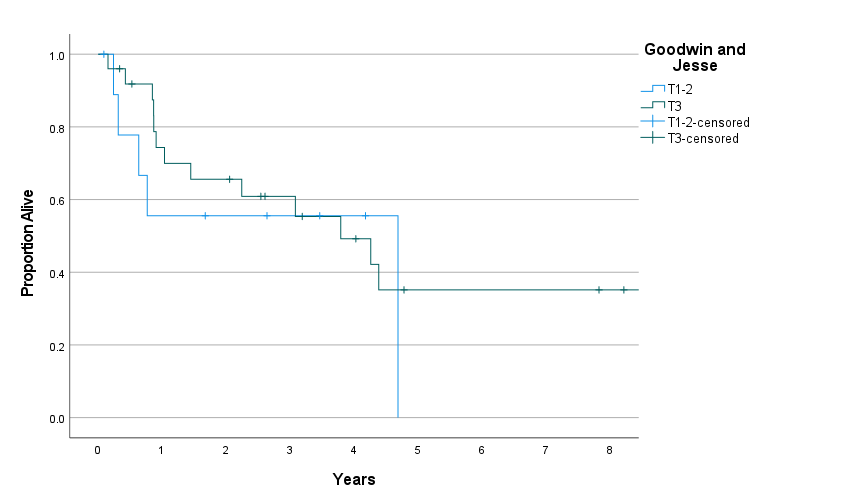


Figure S9. Overall survival of patients with carcinoma of the external auditory canal classified by Crabtree classification.


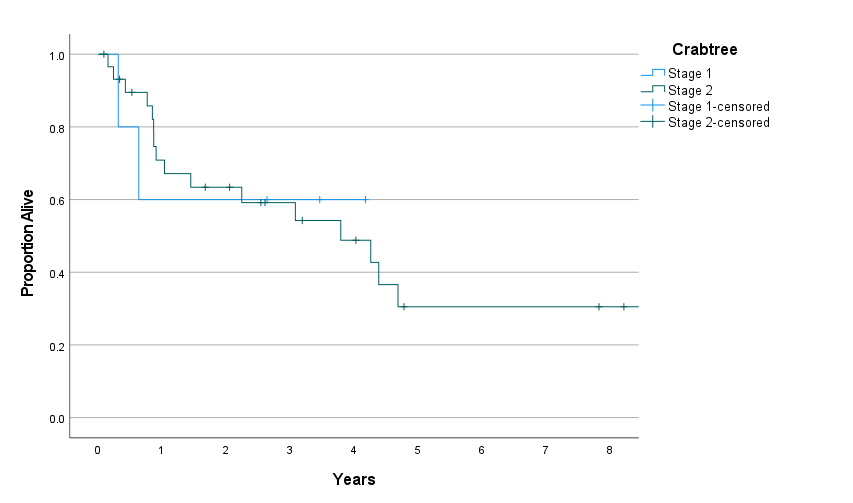


Figure S10. Overall survival of patients with carcinoma of the external auditory canal classified by the presence of regional lymph node.


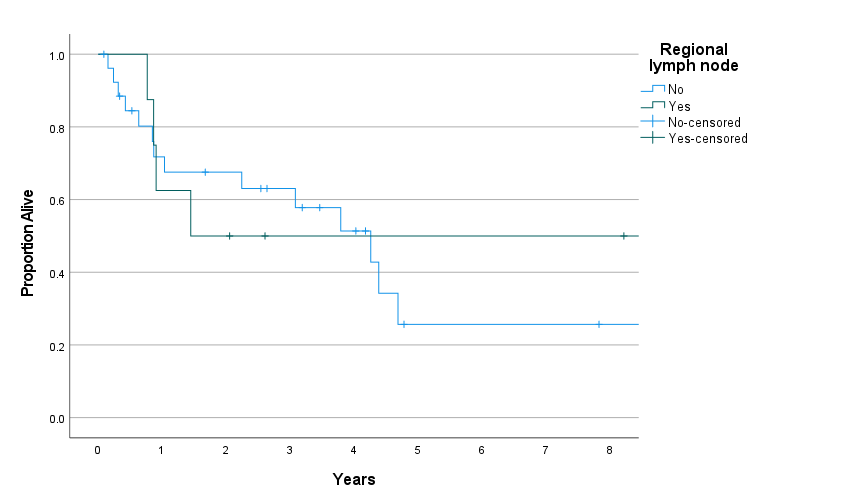


Figure S11. Overall survival of patients with carcinoma of the external auditory canal classified by the presence of cervical lymph node.


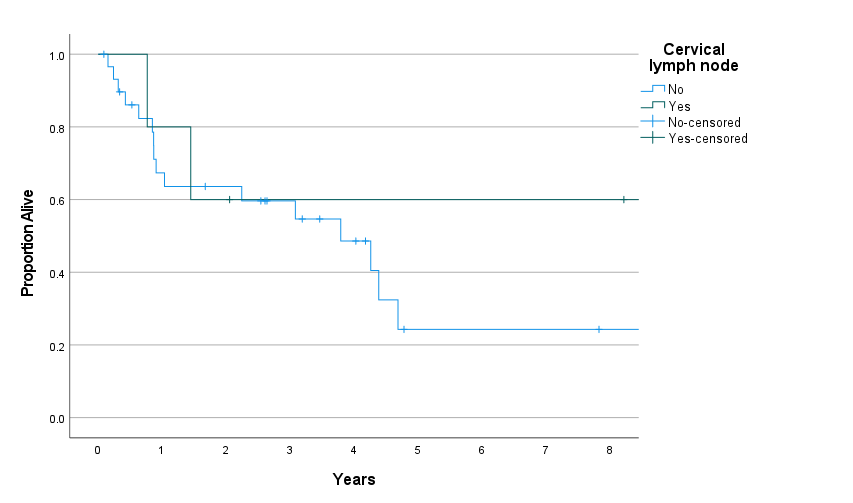

Supplement: Supplementary file 1 — Supplementary Material 1 [file 405_2025_9489_MOESM1_ESM.docx]
