# Supplementary material for: Comparison of 11 staging classifications in carcinoma of the external auditory canal
Source: Eur Arch Otorhinolaryngol. 2025 Jun 2;282(9):4765–71. doi: 10.1007/s00405-025-09489-4 (PMC12423134; doi:10.1007/s00405-025-09489-4)
Supplement: Supplementary file 2 — Supplementary Material 2 [file 405_2025_9489_MOESM2_ESM.docx]

**Table S1. Definitions of selected staging classifications of carcinoma of the external auditory canal**

|  | **Goodwin and Jesse 1980(4)** | **Pittsburgh, Arriaga 1990(2)** | **Lavieille 1997(3)** | **Modified Pittsburgh, Moody 2000(1)** | **AJCC 8th edition Cutaneous carcinoma of the head and neck** | **George 2021(5)** |
| --- | --- | --- | --- | --- | --- | --- |
| T1 | Tumor involved conchal region, pinna or cartilaginous ear canal | Tumor limited to EAC without bony erosion or evidence of soft tissue extension | | | Tumor ≤2 cm in greatest dimension | **A** Tumor limited to EAC with soft tissue involvement |
| T2 | Tumor involved superficial portion of temporal bone (bony ear canal or mastoid cortex) | Tumor with limited EAC bone erosion (not full thickness), or radiographic findings consistent with limited (<0.5 cm) soft tissue involvement, including lesions that extend through preformed pathways such as cartilaginous fissures or bone-cartilage junction of EAC | | | Tumor >2 cm but ≤4 cm in greatest dimension |  |
| T3 | Tumor involved deep structures of temporal bone (middle ear, facial canal, base of skull or mastoid air cells) | Tumor eroding osseous EAC (full thickness) with limited (<0.5 cm) soft tissue involvement, or tumor involving middle ear or mastoid, or **with facial paralysis** | | Tumor eroding osseous EAC (full thickness) with limited (<0.5 cm) soft tissue involvement, or tumor involving middle ear or mastoid | Tumor >4 cm in maximum dimension, minor bone erosion,  perineural invasion or deep invasion* | **B** Tumor eroding full thickness osseous EAC or involving middle ear, mastoid, parotid with or without parotid nodes, infratemporal fossa, temporo-mandibular joint or both (with or without facial palsy) where it's considered still operable |
| T4 |  | Tumor eroding cochlea, petrous apex, medial wall of middle ear, carotid canal, jugular foramen or dura, or with extensive soft tissue involvement (>0.5 cm) | -**T4a** lateral cutaneous tissues (concha, retroauricular skin), parotid structures,  temporomandibular joint or infratemporal fossa involvement  -**T4b** inner ear and petrous apex involvement  -**T4c** dural and intradural involvement | Tumor eroding cochlea, petrous apex, medial wall of middle ear, carotid canal, jugular foramen or dura, or with extensive soft tissue involvement (>0.5 cm), or **with facial paresis** | -**T4a** Tumor with gross cortical bone/marrow invasion  -**T4b** Tumor with skull base invasion and/or skull base foramen involvement | **C** Tumor with intracranial extension |
| N |  |  |  | Lymph node involvement | Regional lymph node involvement | **D** Tumor with cervical node involvement |

*Deep invasion is defined as invasion beyond the subcutaneous fat

or > 6 mm (as measured from the granular layer of adjacent normal epi-

dermis to the base of the tumor); perineural invasion for T3 classification

is defined as tumor cells within the nerve sheath of a nerve lying deeper

than the dermis or measuring 0.1 mm or larger in caliber, or presenting

with clinical or radiographic involvement of named nerves without skull

base invasion or transgression
